# The Congress

**Published:** 1887-11

**Authors:** 


### The Congress.

A large part of the present issue of the Register is devoted to the publication of a report of the proceedings of the Dental Section of the International Medical Congress; and this is only a portion. We hope to give the remainder in the next issue. The report is a long one, but it presents fully and accurately the doings of the section, and is well worthy a careful reading. The publication of the entire proceedings of the congress is being pushed forward as rapidly as possible, and the hope is to have them ready for delivery some time in February next.

These will be sent to all members of the congress in virtue of their membership. They can be obtained by others by sending $10 to Dr. J. B. Hamilton, Washington City, who is editor in chief of the transactions. They will then receive them through the same medium as the members. They will be a very valuable addition indeed to any dentist's or physician's library, worth many times the cost of the books. Only a very limited number will be published in addition to those for the members, so that it will be necessary for all who desire copies to apply early. The price should be sent with the order.

